# PSMA diagnostics and treatments of prostate cancer become mature

**DOI:** 10.1007/s40336-018-0270-2

**Published:** 2018-03-07

**Authors:** Finn Edler von Eyben, Glenn Stewart Baumann, Richard Paul Baum

**Affiliations:** 1 Center for Tobacco Control Research, Birkevej 17, 5230 Odense M, Denmark; 2 Deparment of Radiation Oncology, University of Western Ontario in London, London, ON Canada; 3 Theranostic Center for Radiotherapy and Molecular Imaging, Zentralklinik Bad Berka, Bad Berka, Germany

**Keywords:** Prostate cancer, ^68^Ga HBED-CC PET/CT, ^117^Lu-PSMA-617 RLT

## Introduction

Prostate cancer is the second leading cause of cancer death for men in many parts of the world. Radical prostatectomy (RP) and external beam radiotherapy (EBRT) are effective treatment of localized prostate cancer but many patients recur with raising levels of prostate-specific antigen (PSA). Localized salvage therapies for suspected local failure such as salvage EBRT after RP are most effective during early PSA recurrence. In a Danish national cohort study, salvage EBRT for patients with PSA recurrence after RP undertaken without restaging imaging was effective for approximately half of the patients [1]. Improving on these results requires identification of men with truly localized or oligometastatic recurrence. However, restaging with conventional imaging such as CT and bone scans has limitations. For example, among patients with PSA recurrence after RP, those with a rising PSA < 10 µg/L rarely have positive bone scans. This is problematic because guidelines recommend that patients with PSA recurrence after RP should undergo salvage radiotherapy, while PSA is < 0.5 µg/L [2]. Thus, bone scans cannot guide salvage treatment for most patients with early-phase PSA recurrence after RP.

Prostate membrane antigen (PSMA) is highly expressed in poorly differentiated, metastatic, and castration-resistant prostate cancer. In recent years, there has been enormous progress in the use of PSMA for diagnostics and for treatment with the annual number of pubmed hits for the search words “PSMA” and “prostate cancer” increasing nearly five fold over the last 5 years (from 132 to 630). The challenging question is whether PSMA used for diagnostics and treatment of prostate cancer can postpone progression to death and reduce mortality.

## Restaging with PSMA PET/CT

Use of “the right treatment for the right patient at the right time “may be guided by the right imaging [3, 4]. As documented mainly from Italian departments of nuclear medicine, choline PET/CT has a better sensitivity than conventional imaging [5]. Choline PET/CT detects positive sites for most recurrent patients with PSA > 2 µg/L. However, PSMA PET/CT is significantly more sensitive than choline PET/CT. Thus, an area of research interest is use of PSMA PET for the restaging of men with suspected recurrence after RP or EBRT. PSMA PET/CT is positive for approximately half of the patients with a PSA in the range of 0.5–1.0 µg/L and for more than three quarters of patients with PSA recurrence and PSA > 1 µg/L [6]. As a result, many departments of nuclear medicine that previously used choline PET/CT, switched to PSMA PET/CT.

Current PSMA PET/CT results suggest that restaging localizes the site of recurrence for many recurrent patients with PSA levels of 0.2–0.5 µg/L [2]. That is the range of PSA levels where localized salvage therapies are most effective. In retrospective cohorts, the use of restaging PSMA PET/CT for patients with PSA recurrence after RP changed treatment for up to half of the patients. Now, outcome has been reported for patients with metastases in lymph nodes where restaging PSMA PET/CT guided the salvage treatment. It was salvage lymph node dissection in some studies [7–12] and other studies used salvage EBRT [13–16]. All studies reported a measure of maximal PSMA decline following the salvage treatment and some of the studies also reported the frequency of PSA recurrence-free survival. Studies with longer follow-up will increase the insight of the long-term outcome from salvage treatment guided by restaging PSMA PET/CT.

Use of restaging PSMA PET/CT for patients with PSA recurrence in an early phase may reveal only up to 3–5 positive sites, denoted oligometastatic cancer. Identification and treatment of oligometastatic cancer with targeted therapies such as surgery or EBRT may allow deferral of systemic therapies such as androgen deprivation therapy (ADT), thereby delaying potential morbidity associated with systemic salvage therapy. For example, in a randomized phase 2 study of oligometastatic cancer, compared with surveillance, metastasis directed radiotherapy nearly doubled the survival free from treatment with ADT [17].

Many hospitals use PSMA PET/CT as an advanced imaging modality. Most departments of nuclear medicine in Australia use PSMA PET/CT and so is the case in Germany. In contrast, PSMA PET/CT was not used in France. In USA, the Federal Drug Administration (FDA) approved ^11^C-choline PET/CT for prostate cancer in 2012, and recently also approved anti-1-amino-3-^18^F-fluorocyclobutane-1-carboxylic acid (anti-3-^18^F-FACBC, fluciclovine, ^18^F). Additionally, within the USA and worldwide PSMA PET agents vary with some jurisdictions favoring ^68^Ga-labeled agents and some favoring ^18^F-labeled agents [18]. Each isotope has its advantages and disadvantages. For example, generators needed for ^68^Ga are more widespread than cyclotrons needed for ^18^F, but ^18^F-based PET imaging has higher resolution and contrast than ^68^Ga-based PET imaging.

Future challenges include rigorous, prospective validation of PSMA-based diagnostics such as PET/CT and PET/MRI that will address regulatory and commercialization imperatives to facilitate the routine use. Specialists and societies may continue to work out guidelines for performances and reporting of PSMA PET/CT imaging.

## PSMA-based radioligand therapy

Whereas EBRT targets macrometastases, systemic treatments target both micro- and macrometastases. Drugs such as abiraterone and enzalutamide, inhibiting the androgen receptor pathway, and docetaxel and cabazitaxel, two types of chemotherapy, prolong life for patients with metastatic castration-resistant prostate cancer (mCRPC). But the drugs and even combinations of the drugs do not cure mCRPC. Therefore, PSMA is also used for therapy. As opposed to detection with PSMA PET/CT imaging, small molecule binding to PSMA can be linked by a chelator to a therapeutic isotope to treat cancer lesions in a theranostic approach. The most reported treatment using PSMA is ^177^Lutetium-PSMA-617 radioligand therapy (RLT). Initially, ^177^Lu-PSMA-617 RLT was used for patients with PSMA PET/CT-positive mCRPC who had failed to the life-prolonging drugs. For such patients, ^177^Lu-PSMA-617 RLT often gave relief of pain from bone metastases, decline of PSA, and objective remission with long progression-free survival and even prolongation of survival [19]. The treatment gave mainly minor adverse effects. A recent meta-analysis compared findings with ^177^Lu-PSMA-617 RLT and third-line treatment [20]. In two of three effect endpoints ^177^Lu-PSMA-617 RLT gave better outcome than third-line treatment. Third-line treatment with cabazitaxel caused more grade 3 and 4 adverse effects than ^177^Lu-PSMA-617 RLT.

Despite these encouraging results, the use of ^177^Lu-PSMA-617 RLT has not yet reached the point of being considered a standard treatment option. For example, the second Advanced Prostate Consensus Conference (APCCC) in St Gallen 2017 did not endorse ^177^Lu-PSMA-617 RLT [21]. APCCC is a forum of expert’s opinion for questions of management of advanced prostate cancer that at present is unsolved by randomized trials. APCCC 2017 argued that the treatment mainly had been reported in small series from single hospitals. APCCC 2017 also argued that ^177^Lu-PSMA-617 RLT should only become a standard treatment option if phase 3 trials showed the treatment had an advantage over standard care. The rapid incorporation of radium-223, targeting only bone metastases, as a standard treatment option for men with mCRPC based on the prolongation of life in a randomized phase 3 trial [22] suggests that similar trials soon should be conducted for PSMA-based RLT that targets PSMA-PET/CT-positive sites in bones as well as elsewhere. Fortunately, such prospective trials are now commencing.

## PSMA in future

Despite the evidence accumulated to date, the role of PSMA in personalizing prostate cancer treatment remains the subject of many clinical trials. For example, Clinicaltrials.gov reports that 24 of 61 trials of PET in prostate cancer are based on PSMA. Along with the PSMA trials are trials of other imaging probes such as choline (6 trials), sodium fluoride (1 trial), FACBC (3 trials), other markers such as FLT, fluoro-deoxy-glucose (FDG), miso, acetate, and citrate (6 trials), and novel probes (7 trials). Thus, the landscape of prostate cancer imaging and characterization continues to evolve and expand, but PSMA will undoubtedly achieve a dominant role in diagnostics of prostate cancer.

As of January 2018, the Australian and New Zealand Urogenital and Prostate Cancer Trial Group started a prospective multicenter randomized phase 2 study (ClinicalTrials NCT 03392428). The study uses two arms of monotherapy. The study compares second- and third-line therapy of mCRPC with either ^177^Lu-PSMA-617 RLT or cabazitaxel. ^177^Lu-PSMA-617 RLT is given in cycles with 6 weeks intervals and injecting 8.5 GBq of ^177^Lu PSMA-617 in the first cycle, de-escalated to 6.0 GBq in cycle 6. The dose of cabazitaxel is 20 mg/m^2^ body surface given with 3 week intervals. That is the schedule recommended by APCCC 2017. The study intends to recruit two hundred patients and aims to investigate whether ^177^Lu-PSMA-617 RLT will give a higher frequency of PSA decline ≥ 50% than cabazitaxel. In case the randomized trial confirms that ^177^Lu-PSMA-617 RLT has an advantage relative to third-line treatment with cabazitaxel, the outcome represents an external validation of the previous meta-analysis [20]. Such a consistency may lead to a paradigm shift regarding standard treatment of patients with mCRPC who progress after first- and second-line treatment.

The consistency also encourages exploration of RLT in early stages of recurrent prostate cancer. The Prostate Cancer Clinical Trials Working Group (PCWG) argued that treatment that had shown effective for end-stage patients should be tested for a possible role at an early stage of the cancer [23]. Reviews of ^177^Lu-PSMA-617 RLT agree that the treatment is effective for end-stage patients. However, the Australian randomized trial evaluating ^177^Lu-PSMA-617 RLT as second- or third-line treatment does not study prostate cancer in an early stage. Thus, further phase 3 trials are warranted. In accordance, phase 3 trials evaluating the role of ^177^Lu-PSMA-617 RLT for patients in an early phase of prostate cancer are being planned. Prospective studies are also required for optimization of PSMA-based RLT through selection of optimal agents and dosing schedules.

## Conclusion

PSMA-based PET/CT imaging is increasingly being implemented in many countries and PSMA theranostics are emerging as highly promising tools in the treatment of mCRPC. Randomized trials are now underway to elucidate whether PSMA used for diagnostics and treatment of prostate cancer can postpone progression to death and reduce mortality. That may lead to revision of present guidelines for PSMA-based PET/CT and ^177^Lu-PSMA-617 RLT. That might also lead to its incorporation into the present sequence of treatments for mCRPC. The next APCCC conference in 2019 might give a revised consensus statement regarding PSMA for diagnostics and treatment of advanced prostate cancer.

## References

[1] Ervandian M, Hoyer M, Petersen SE, Sengelov L, Hansen S, Holmberg M, Veistrup, Maidahl Petersen P, Borre M (2016). Salvage radiation therapy following radical prostatectomy. A National Danish study. Acta Oncol.

[2] Pfister D, Bolla M, Briganti A, Carroll P, Cozzarini C, Joniau S, van Poppel H, Roach M, Stephenson A, Wiegel T, Zelefsky MJ (2014). Early salvage radiotherapy following radical prostatectomy. Eur Urol.

[3] Baum RP, Rösch F, 1st World Congress on Ga-68 and Peptide Receptor Radionuclide Therapy (PRRNT), June 23–26, 2011, Zentralklinik Bad Berka, Germany. World J Nucl 10 PubMed PMID: 22034575 10.4103/1450-1147.82105 PMC3198045 22034575

[4] von Eyben FE, Kiljunen T, Kangasmaki A, Kalevi K, von Eyben R, Joensuu T (2016). Management of prostate cancer. Ann Oncol.

[5] von Eyben FE, Kairemo K (2014). Meta-analysis of (11)C-choline and (18)F-choline PET/CT for management of patients with prostate cancer. Nucl Med Commun.

[6] von Eyben FE, Picchio M, von Eyben R, Rhee H, Bauman G (2016). ^68^Ga-labeled prostate-specific membrane antigen ligand positron emission tomography/computed tomography for prostate cancer: a systematic review and meta-analysis. Eur Urol Focus.

[7] Hijazi S, Meller B, Leitsmann C, Strauss A, Meller J, Ritter CO, Lotz J, Schildhaus HU, Trojan L, Sahlmann CO (2015). Pelvic lymph node dissection for nodal oligometastatic prostate cancer detected by 68 Ga-PSMA-positron emission tomography/computerized tomography. Prostate.

[8] Herlemann A, Kretschmer A, Buchner A, Karl A, Tritschler S, El-Malazi L, Fendler WP, Wenter V, IIhan H, Barenstein P, Stief CG (2017). Salvage lymph node dissection after (68)Ga-PSMA or (18)F-FEC PET/CT for nodal recurrence in prostate cancer patients. Oncotarget.

[9] Montorsi F, Gandaglia G, Fossati N, Suardi N, Pultrone C, de Groote R, Dovey Z, Umari P, Gallina A, Briganti A, Mottrie A (2017). Robot-assisted salvage lymph node dissection for clinically recurrent prostate cancer. Eur Urol.

[10] Porres D, Pfister D, Thissen A, Kuru TH, Zugor V, Buettner R, Knuechel R, Verburg FA, Heidenreich A (2017). The role of salvage extended lymph node dissection in patients with rising PSA and PET/CT scan detected nodal recurrence of prostate cancer. Prostate Cancer Prostatic Dis.

[11] Rauscher I, Duwel C, Wirtz M, Schottelius M, Wester HJ, Schwamborn K, Haller B, Schwaiger M, Geschwend JE, Eiber M, Maurer T (2017). Value of (111) In-prostate-specific membrane antigen (PSMA)-radioguided surgery for salvage lymphadenectomy in recurrent prostate cancer: correlation with histopathology and clinical follow-up. BJU Int.

[12] Siriwardana A, Thompson J, van Leeuwen PJ, Doig S, Kalsbeek A, Emmett L, Delprado W, Wong D, Samaratunga H, Haynes AM, Coughlin G, Stricker P (2017). Initial multicentre experience of (68) gallium-PSMA PET/CT guided robot-assisted salvage lymphadenectomy: acceptable safety profile but oncological benefit appears limited. BJU Int.

[13] Emmett L, van Leeuwen PJ, Nandurkar R, Scheltema MJ, Cusick T, Hruby G, Kneebone A, Eade T, Fogarty G, Jagavkar R, Nguyen Q, Ho B, Joshua AM, Stricker P (2017). Treatment outcomes from (68)Ga-PSMA PET/CT-informed salvage radiation treatment in men with rising PSA after radical prostatectomy: prognostic value of a negative PSMA PET. J Nucl Med.

[14] Guler OC, Engels B, Onal C, Everaert H, Van den Begin R, Gevaert T, de Ridder M (2017). The feasibility of prostate-specific membrane antigen positron emission tomography(PSMA PET/CT)-guided radiotherapy in oligometastatic prostate cancer patients. Clin Transl Oncol.

[15] Henkenberens C, von Klot CA, Ross TL, Bengel FM, Wester HJ, Katja H, Christiansen H, Derlin T (2017). 68 Ga-PSMA ligand PET/CT-based radiotherapy for lymph node relapse of prostate cancer after primary therapy delays initiation of systemic therapy. Anticancer Res.

[16] Zschaeck S, Wust P, Beck M, Wlodarczyk W, Kaul D, Rogasch J, Budach V, Furth C, Ghadjar P (2017). Intermediate-term outcome after PSMA-PET guided high-dose radiotherapy of recurrent high-risk prostate cancer patients. Radiat Oncol.

[17] Ost P, Reynders D, Decaestecker K, Fonteyne V, Lumen N, De Bruycker A, Lambert B, Derue L, Bultijnck R, Clayes T, Gothghebeur E, Villeirs G, De Man K, Ameye F, Billiet I, Joniau S, Vanhaverbeke F, De Meerleer G (2018). Surveillance or metastasis-directed therapy for oligometastatic prostate cancer recurrence: a prospective, randomized, multicenter phase II trial. J Clin Oncol.

[18] Evans JD, Jethwa KR, Ost P, Williams S, Kwon ED, Lowe VJ, Davies BJ (2018). Prostate cancer-specific PEwester HT radiotracers: a review on the clinical utility in recurrent disease. Pract Radiat Oncol.

[19] Baum RP, Kulkarni HR, Schuchardt C, Singh A, Wirtz M, Wiessalla S, Schottelius M, Klette I, Wester HJ (2016). 177Lu-labeled prostate-specific membrane antigen radioligand therapy of metastatic castration-resistant prostate cancer: safety and efficacy. J Nucl Med.

[20] von Eyben FE, Roviello G, Kiljunen T, Uprimny C, Virgolini I, Kairemo K, Joensuu T (2018). Third-line treatment and (177)Lu-PSMA radioligand therapy of metastatic castration-resistant prostate cancer: a systematic review. Eur J Nucl Med Mol Imaging.

[21] Gillessen S, de Bono JS, Sartor O, Omlin AG (2017) Reply to Finn E. von Eyben, Irene Virgolini and Giandomenico Roviello’s Letter to the Editor re: Silke Gillessen, Gerhardt Attard, Tomasz M. Beer, et al. Management of Patients with Advanced Prostate Cancer: The Report of the Advanced Prostate Cancer Consensus Conference APCCC 2017. Eur Urol. In press. Eur Urol 2017. http://dx.doi.org/10.1016/j.eururo.2017.06.002

[22] Parker C, Nilsson S, Heinrich D, Helle SI, O’Sullivan JM, Fossa SD, Chodacki A, Wieckno P, Logue J, Seke M, Widmark A, Johannessen DC, Hoskin P, Bottomley D, James ND, Solberg A, Syndikus I, Kliment J, Wedel S, Boehmer S, Dall’Oglio M, Franzen L, Coleman R, Vogelzang N, O’Bryan-Tear CG, Stadacher K, Garcia-Vargas J, Shan M M, Bruland Ø, Sartor O, AL SYMPCA Investigators (2013). Alpha emitter radium-223 and survival in metastatic prostate cancer. N Engl J Med.

[23] Scher HI, Eisenberger M, D’Amico AV, Halabi S, Small EJC, Soule HR, Morris M, Kattan MW, Roach M, Kantoff P, Pienta KJ, Crducci MA, Agus D, Slovin SF, Heller G, Kelly WK, Lange PH, Petrylak D, Berg W, Higano C, Wilding G, Moul JW, Partin AN, Logothesis C, Soule HR (2004). Eligibility and outcomes reporting guidelines for clinical trials for patients in the state of a rising prostate-specific antigen: recommendations from the Prostate-Specific Antigen Working Group. J Clin Oncol.

